# The imagined scientist of science governance

**DOI:** 10.1177/0306312720962573

**Published:** 2020-10-17

**Authors:** Heidrun Åm, Gisle Solbu, Knut H Sørensen

**Affiliations:** 1 Department of Sociology and Political Science, Norwegian University of Science and Technology, Norway; 2 Department of Interdisciplinary Studies of Culture, Norwegian University of Science and Technology, Norway

**Keywords:** biotechnology, deficits, imagined scientist, nanotechnology, science policy, responsible research and innovation

## Abstract

In this article, we introduce the concept of ‘the imagined scientist’. It inverts previous discussions of the public as an imagined community with a knowledge deficit, to examine imagined scientists representing an actor (or group of actors) with deficits in knowledge or concern about social issues. We study how Norwegian science policymakers, on the one hand, and biotechnologists and nanotechnologists, on the other, articulate and engage with social responsibility. The article identifies what we call ‘deficit trouble’, when there is poor alignment of the deficits of different imagined scientists, which may lead to a stalemate in the communication between science policymakers and scientists. We argue that ‘the imagined scientist’ can function as sensitizing concept for further studies of science governance across a range of topics, bringing into view how different deficit logics operate in science policy.

## Introduction: A new approach to study science governance

Science policy draws on representations of scientists and their practices, to shape governance efforts, incentive structures, allocation of resources, knowledge mobilization and more. For example, the widespread establishment of institutions to ensure that scientists adhere to research ethics is based on policymakers’ belief that if scientists are left to their own devices, they will be tempted to participate in unethical practices (Hilgartner et al., 2017). In addition, scientists, when prompted, may offer representations of themselves and the features that constitute a ‘proper’ scientist. The underlying ideas may influence how scientists engage with science policy and science governance, including their interaction with science policymakers. In this article, we analyze science policymakers and scientists’ discursive constructs of scientists and their practices.

We introduce the concept of ‘the imagined scientist’ to the study of science governance. Our conceptual work is inspired by studies of how scientists conceive of their publics. These studies use concepts such as the imagined layperson or the imagined publics (e.g. Heidenreich, 2015; Maranta et al., 2003; Solbu, 2018; Walker et al., 2010; Welsh and Wynne, 2013). The premise of this scholarship is that imagined publics are performative in the sense that such conceptions may influence scientists’ research and forms of engagement. Similarly, we assume that ‘the imagined scientist’ is performative with respect to shaping and mobilizing science governance.

Our analysis of the imagined scientist centers on articulations of the social responsibility of science and its attendant issues. The concept of social responsibility is a multi-faceted term used to focus on a wide set of concerns, such as usability, social relevance, public participation and the moral accountability of science. The concern for usefulness was important to the development of the modern research university (Clark, 2007), while scientists’ moral accountability emerged as an important issue after WWII, demonstrated, for example, by the establishment of the *Bulletin of Atomic Scientists* in 1945, and, more recently, the March for Science.

There has also been a growing interest in social responsibility issues in ongoing debates about science policy. A pertinent example is the policy initiative of Responsible Research and Innovation (RRI) that receives considerable attention in the Horizon 2020 program of the European Union (EU) and elsewhere (e.g. Stilgoe and Guston, 2017). In this article, we use such RRI initiatives as points of departure for studying how scientists’ conceive of their self-conduct; juxtaposing their imagined scientist with science policy’s imagined scientist. In our analysis, we employ an open-ended understanding of social responsibility of science, in which we empirically identify actors’ expressions of virtues and deficits regarding science-society concerns. It is important to notice that we use the imagined scientist and his/her deficits as analytical categories that are not actively used by the actors we study. Rather, these are concepts that we, as social scientists studying science policies and social responsibility, employ to study sense-making in science policy implementation.

In the following, we first analyze the views of science policymakers as they are expressed through documents outlining RRI in the context of the Research Council of Norway. Second, we examine interviews with bio- and nanotechnology scientists in which they provide accounts of how they understand and manage social responsibility issues in their work. We then discuss the different constructions of imagined scientists, with a focus on the assumed distribution of social responsibility among science policy actors. Often, RRI scholars confer this responsibility onto scientists. However, they may also direct it toward universities, funding institutions, policymakers and industry (e.g. Owen et al., 2012). This projecting and shifting of responsibility introduce an interesting ambiguity between institutional and individual responsibility that we also investigate through the analysis of ‘the imagined scientist’.

## Images of scientists: Institution, practice and deficits

There is a widespread view that scientists reside in a metaphoric ivory tower, disengaged from society. Shapin (2012) critically analyses the emergence of this stereotype. Based on his study of scientific work in the 21st century, he argues that scientists are deeply engaged in socially relevant issues. A growing body of research supports this claim. For example, McCarthy and Kelty (2010) and Kjølberg and Strand (2011) have analyzed scientists working in nanotechnology and found that they actively dealt with ethical, legal and social aspects of their research. Other studies have focused on the management of science-society relations through ethics, which has become an important element of science governance (Braun et al., 2010; Salter and Salter, 2007; Tallacchini, 2015). In this article, we use the concept of the imagined scientist as a tool to study empirically how science policymakers, on the one hand, and bio- and nanotechnology scientists, on the other, articulate how scientists engage with science-society relations.

As noted, the introduction of ‘the imagined scientist’ is inspired by previous research on so-called imagined publics or imagined laypersons in the context of public understanding of/engagement with science and technology. Initially, this research analyzed the content and consequences of scientists’ deficit model of the public understanding of science and technology – the view that negative public responses to scientific claims or to technological changes are due to public deficiencies in the understanding of science (Irwin and Wynne, 1996). For example, Maranta et al. (2003) explore discursive constructs of an unknowledgeable public and their effects on scientists’ practices. Later work has focused on how scientists describe what scholars usually call public knowledge deficits and the tendency to consider such deficits as potentially harmful. Many scientists have been observed to worry that knowledge deficits may produce public skepticism and resistance towards new forms of science and technology (e.g. Barnett et al., 2012; Walker et al., 2010). However, the characteristics of such imagined publics vary. For example, Heidenreich (2015) offers observations of little concern for deficits among the scientists she studied.

We flip the idea of a public knowledge deficit to ask if some form of deficit is an integral part of science governance’s imagined scientist. At least some RRI scholars suggest that scientists lack knowledge about social responsibility and about how to address concerns about it, and that such deficits may affect the public’s trust in science and innovation (e.g. Owen et al., 2012; Stilgoe and Guston, 2017; Stilgoe et al., 2013). However, these scholars do so without implying that scientists are skeptical of or resist demands for an increased focus on exercising social responsibility in their research. Overall, deliberations regarding the social responsibility of science may reflect distinct images of how science should be done, as well as of what scientists should care about and be capable of doing with respect to science-society relations. We explore such images by analyzing how the two sets of actors that we study describe scientists and their doings in the context of science-society relations.

We focus on and classify the imagined deficits invoked by the different parties. In this, we take inspiration from a recent analytic framework of how deficit logics operate, developed by Pfotenhauer et al. (2019) in their investigation of innovation policies. Like us, they draw on studies of imagined publics, while arguing that a focus on deficit logics in the context of public policy does not raise the same problems as in the context of public understanding of science. The concept ‘deficit logic’ points to the performativity of thinking in terms of deficits. Pfotenhauer et al. suggest five dimensions to systematize various layers of deficit constructions and effects: (1) problem diagnosis (what kind of deficit is being diagnosed), (2) proposed solution (suggested remedies to address deficit), (3) source of expertise (expertise considered legitimate to address deficits), (4) social order (orders implied in solutions), and (5) standard for success (p. 896). In our study, we analyze the construction of such deficit logics at two sites, including what kind of knowledge about scientists’ work that is used.

We do not make a priori assumptions about how the imagined scientist and their related deficits are or should be articulated at the two sites we study. An open-ended concept of deficit such as the one proposed by Pfotenhauer and colleagues, is useful to our investigation because it provides a conceptual tool to empirically characterize the imagined scientist of the two sites we study. Our refined main research questions are thus: How do science policymakers and scientists imagine scientists’ knowledge (or knowledge deficits) and agency in relation to the enactment of social responsibility in and knowledge about science-society relations? What suggested solutions follow from these problem framings? To what extent are these characteristics drawing on knowledge about the culture and practice of science? To what extent are the ensuing ‘imagined scientists’ aligned? In our analysis, we first explore our data by pursuing these questions, and then discuss our findings by employing the deficit framework presented above.

## Method

As already noted, the article draws on two empirical sources. The first is group of RRI-related documents from the Research Council of Norway (RCN), which is the only significant research funding organization in Norway. The RCN thus has a broad disciplinary and cross-sectorial reach and is positioned prominently vis-à-vis the government; it is also its official science policy advisor. We chose to study these RRI documents because they are official articulations of how the most prominent science policy-making institution in Norway constructs an image of scientists and simultaneously emphasizes a particular understanding of social responsibility through the identification of particular deficits.

The RCN’s policy documents, such as its RRI framework, do not represent only the points of view of isolated administrators. The articulation of science policy is usually a networked achievement (e.g. de Saille, 2015). These networks consist of epistemic communities where scientists, administrators, technical experts, governmental actors, and sometimes citizens and others, interact and exchange views; representatives of scholarly fields such as STS, philosophy and ethics also play important roles. However, when scientists provide inputs to policymaking, they enact other roles than when they do research.

The main document for our analysis was the RCN’s so-called RRI framework report, which aims to clarify the RRI concept to scientists who are required to incorporate some form of RRI into their research proposals (RCN, n.d.). The four-page document also explains the governance context and the motives for introducing RRI, and it suggests how to incorporate the concept to change scientific practices. In addition, we studied work plans and strategy documents related to RCN’s bio- and nanotechnology programs, their grant calls and application forms, and RCN’s overall strategy plan. Because RCN’s pursuit of RRI policies has mainly been implemented in these two programs, the RRI framework document has affected most tangibly nano- and biotechnology scientists since they have been obliged to show in their funding applications how they plan to implement the framework in their projects. With respect to biotechnology, we have also studied the documents leading to the establishment of the national research center Digital Life Norway, which gave RRI distinct attention. Thus, the RRI framework document provides not just advice but also a policy that has been realized, most evidently in the grant application forms of the RCN. We have also observed this through our recurrent interaction with RCN actors.

All the documents engage in discursive constructions of scientists. To elicit these constructions, we began by posing four questions in the analysis of the texts: (1) How do the documents define the problems to be targeted through RRI efforts? (2) Whom do the documents identify as the main actors to be involved in these efforts? (3) How do they describe scientists as part of RRI efforts? (4) What do they present as obstacles for RRI? Through these questions, we identify explicit and implicit imagined deficits related to social responsibility and the consequent features of solutions to these deficits.

To elicit scientists’ own ‘imagined scientist’, we interviewed 37 scientists in bio- and nanotechnology working in Norway. We selected scientists from these fields of emerging technologies because, as we observe above, they are prominent in the analyzed RCN documents. In the interviews, we asked questions pertaining to the social relevance and benefits for society of the scientists’ work, including whether they considered being part of solving so-called grand challenges through their research. We inquired about what interviewees were doing to make research results useful and in what way they anticipated how their research would affect society, including potential harms. Further, we asked them to describe concrete incidents where they had experienced ethical, legal, or social challenges and how they managed these. In this manner, we mapped what the interviewees considered social responsibility and respective appropriate self-governance.

Seven of the interviews were conducted in English, the rest in Norwegian. All interviews were taped, transcribed and anonymized. Unless the interview was conducted in English, quotes have been translated from Norwegian by the authors. The interview data were analyzed and coded manually using the computer software Atlas.ti. This coding aimed at identifying discursive constructs of scientists and their practices, articulated deficits, and proposed solutions to close these deficits related to science-society relations. We also coded information about the sources used to articulate features of imagined scientists and implied social orders, by both the documents and the interviewees.

The imagined scientist of the policymakers was elicited from the documents as a discursive construct. With the interviewed scientists, the analytical process was somewhat different, since we departed from the interviewees’ accounts of their practices. Still, these accounts tended to imply a normative construct of how scientists ought to enact science-society relations with respect to social responsibility. Accordingly, we see the accounts as a valid source from which to construct the imagined scientist of the scientists. However, it should be noticed that the result is a composite subject, since we had to combine and integrate their accounts, which displayed both heterogeneity and consistency.

## The imagined scientist of Norwegian RRI policy

We analyzed the RCN documents to elicit the underlying approach to science governance (Glerup and Horst, 2014), including problem diagnosis, suggested solutions, actors identified as main agents, and knowledge about research practices and existing social responsibility management. As expected, these documents were informed by the international RRI literature. A striking feature of RRI scholarship (Owen et al., 2012; Stilgoe et al., 2014) is its emphasis on science for the public good through broad involvement of stakeholders. This turn to deliberative technology assessment emerged about 20 years ago, in the aftermath of GMO protests and in the context of nanotechnology governance. Since then, in science governance it is no longer sufficient only to assess the technical and scientific qualities of emerging technologies; safety, sustainability and social issues should also be considered. Public engagement, as well as research on ethical, legal, and social aspects (ELSA) of science and innovation, became an expected feature in policymaking related to technoscientific governance (Irwin, 2006). This assessment regime (Kaiser et al., 2010) invites the promotion of change in the roles of scientists in science governance and practice.

Generally, the analyzed RCN documents diagnosed two problem spaces with respect to social responsibility: (1) a deficit in the extent to which Norwegian researchers have addressed societal challenges, and (2) a knowledge deficit among the scientists regarding the increasing complexity of science-society relations and its consequences for their work. The first of these spaces emanates from broad concerns about scientists’ choice of focus in their research; it is insufficiently oriented towards what RCN actors see as pressing problems. The RCN documents see the two problem spaces as related. The deficit in addressing societal challenges is at least partly caused by the lack of knowledge about science-society relations: Researchers should be more attuned to social needs, which requires a better understanding of what these needs are.

The analyzed documents highlight the useful roles that research should play in Norwegian society, as well as RCN’s mission to oversee the achievement of this aim. The RRI framework document articulates this narrative already in its first paragraph:

… the Research Council must assume greater social responsibility by contributing to make research and innovation provide long-term benefits for society. This means both that research should be conducted in a socially responsible way, and that greater importance is attached to how research might contribute to solving the grand societal challenges. (RCN, n.d.: 1)

The RCN document states that the Council will be in charge of making research and innovation beneficial to the broader society, stipulating that it should be actively involved in ensuring that research addresses the main objectives – ‘the grand challenges’ – formulated by the Norwegian government (RCN, 2015: 1–3). The work plans of the bio- and nanotechnology programs also present social responsibility as an overarching objective. They emphasize the importance of research adhering to official ethical, cultural, health and environmental criteria for the betterment of society. For example, the biotechnology program’s plan document states as one of its six main objectives that it should: ‘Ensure the responsible development of technology that addresses global social challenges in the areas of health and sustainable food and industrial production’ (RCN, 2018: 9). In a similar vein, the nanotechnology program’s main goal is to ‘maximize the technology’s positive contribution to society’s needs and efforts to minimize potential unintended negative impacts on the individual and society at large’ (RCN, 2019: 29). The implied deficits in the texts refer to unrealized potential of research to provide benefits to society and for improvement concerning adherence to social values.

On the second dimension, the complexity issue, the documents diagnose a deficit in the coordination across R&D actors.

Research interacts and is interwoven with other social, cultural and historical factors. The intermingling, complexity and dynamics of this co-production means that governance schemes based on distance and clear task distribution between research, technology, innovation and policy are unproductive. It is in recognition of this systemic complexity and dynamics that the vision of Responsible Research and Innovation has emerged. (RCN, n.d.: 1)

The biotechnology work plan takes this argument further by arguing that scientists need to be more deeply engaged in managing complex science-society relations. First, the plan acknowledges the importance of paying attention to ethical, social and legal aspects to be able to map the future consequences of new biotechnological developments. Second, it states the need for biotechnology researchers to acknowledge that ‘basic, translational and applied research and direct implementation of research results are much more intertwined with each other and have a far more complex relationship to society and production processes in general than has previously been understood’ (RCN, 2019: 29).

Implicitly, the document argues that R&D actors have failed to grasp the complexity of the ‘ecology’ of science-innovation interactions. The document considers that the main effect of the assumed deficit in understanding complex science-society relationships is that it raises a barrier to realizing the full potential of science to be useful to society, including solving grand challenges. In this way, the document points to a wide-ranging deficit with respect to practices of acquiring systemic knowledge of, reflecting about and engaging more actively in science-society relationships.

Who is expected to do the work of enacting social responsibility in terms of addressing societal challenges and coordinating actors? The analyzed documents mention several options, including that the RCN itself should play an important role and form the hub of the wheel. However, in the final instance, the documents expect scientists to be the main contributors. For example, the Norwegian government’s nanotechnology strategy states that ‘scientists and research organizations should be held accountable for the social, environmental and health-related effects of their work’ (Norwegian Government, 2012: 50). In a similar vein, in a public statement about RRI, senior advisors of the RCN highlight that they will implement RCN’s social responsibility by building capacities among young scientists, thus transferring agency to future generations of researchers:

The experience from the RRI work in RCN so far shows that continued development [pursuing RRI virtues] presupposes the building of knowledge, competence, skills and capacity – on an individual as well as on an institutional level. In light of this, the RRI activities hereafter will have a particular orientation towards the Ph.D. students of the research programs. (Gulbrandsen and Rynning, 2016: 27)

This stated orientation of the RCN towards scientists as the primary actors to pursue social responsibility of science is significant.

Apart from that, the analyzed documents provide only quite general statements about the characteristics of social responsibility. The RCN leaves these to the scientists to clarify, at least in their grant applications. For example, the analyzed documents state that scientists shall pursue the four dimensions of RRI emphasized in the RCN’s framework: (1) to anticipate, (2) reflect, (3) engage and (4) adapt. Thus, the RRI framework document suggests an image of scientists with a deficit regarding knowledge, competence and capacity for anticipation, reflection and inclusion. In addition, the RRI framework document asserts that scientists lack knowledge of and humility with respect to the limits of their own knowledge.

[After a period of public dialogue focusing on citizen conferences, etc.], the focus is now increasingly on the research communities themselves. There is a lack of skills related to opening up research and innovation processes, recognizing boundaries for one’s own knowledge and expertise, and being able to ask for help with the possible terrain-changing effects of the [science and technology development] processes. (RCN, n.d.: 3)

In other words, the RCN constructs scientists as powerful actors with potential to change the world and deeply affect society. While the RCN documents also state that social responsibility requires ‘distributed governance orders’ (RCN, n.d.: 3), the responsibility to initiate the invitation of other social actors is ascribed to the imagined scientist. Consequently, the RRI framework document suggests that the imagined scientist needs to become more concerned with anticipating the outcome of the performed research and its impact on other actors. Further, the imagined scientist is expected to reflect and discuss research in a broad sociotechnical context, to open research and innovation efforts to inclusive social dialogues, and to improve the understanding of the limitations of the acquired knowledge and competence. The latter includes recognizing the potential need to ask for assistance in the pursuit of social responsibility.

The RCN documents give institutions such as universities and research institutes an unclear and marginal role. Rather, as we have seen, the documents target individual scientists, focusing on their deficient pursuit of reflexive capacity and socially responsible practices, which are considered to be key to change the conduct of research to comply with RRI goals. Thus, the imagined scientist of the analyzed policy documents does not in general lack social responsibility, as this feature is understood by the RCN. However, the imagined scientist has serious deficits with respect to knowledge about what social responsibility means in the context of the performed research, as well as the competence, skills and capacity to enact the social responsibility requirements of RCN. Still, the imagined scientist was assumed to be able to improve without much outside intervention other than the disciplining efforts made for example through grant proposal templates. Paradoxically, it is left to the uninformed imagined scientist to define what the RRI framework document should mean in practice (Delgado and Åm, 2018). Thus, there is no clear measure of success (Pfotenhauer et al., 2019: 896).

## The imagined scientist of Norwegian bio- and nanotechnology scientists

To what extent do the RCN’s deficit constructions echo views among scientists? Above, we presented the RCN’s ambivalent construction of deficits and the capacity of scientists to pursue RRI. At the outset, we assumed at least some degree of alignment between the imagined scientist of the interviewed scientists and the imagined scientist of the policy documents, given the RCN’s access to disciplining instruments such as grant application requirements. However, we also expected suggestions of an oppositional construct not aligned with the one observed from the RCN documents. In the following, we present scientists’ deficit constructions as they were articulated in our interviews. We structure the presentation according to the deficit logic employed by the policy documents analyzed above. Thus, we first focus on the scientists’ imagined contribution or lack thereof to society. Second, we assemble scientists’ construction and assessment of their science-society knowledge and their suggestions of deficits.

### Being useful

The RCN documents stress that the research funded by the Council should be useful and should help solve so-called grand challenges. We interpret this to imply that the imagined scientist of these documents insufficiently pursues usefulness to society and lacks knowledge about how to become more useful. The documents ask that scientists make more concerted efforts to alert society about the potential benefits of their research, as a strategy for improving its usefulness and quickening the process of reaping benefits. How did the interviewees consider the usefulness of science? Below, we provide a heterogeneous, composite imagined scientist, based on the constructions of our interviewees.

The scientists’ concrete articulations were diverse, reflecting differences between their fields of research and in degrees of orientation towards applications. For example, those who worked on topics related to medicine often described their usefulness with reference to patients.

I’m working towards concrete applications. In my case, these are applications with regard to health. All my projects are about health; this is what is driving me. I feel it’s very rewarding to work to achieve something that can be applied in health-related efforts, improve the daily life of patients, the patients’ families, doctors, right? (IW22)

IW30 expressed a similar intention to be useful in terms of disease treatment. He recently had shifted his research focus. ‘I’ve set myself the goal to help at least one patient before I retire.’ Both these quotes show that being useful in a concrete way was considered important and meaningful. Such motives would also shape how the scientists conceived of and designed their projects. Scientist IW22 was an example, collaborating with medical practitioners to obtain first-hand knowledge about specific problems that needed to be solved. IW23 was another. She changed her research focus from one form of cancer to another to improve the chances of successful clinical trials and to help patient groups. Thus, there was an outspoken commitment to the provision of social benefits.

Other interview accounts of usability showed a similar commitment, but towards industrial justifications of worth that focused on efficiency and industry collaboration (Sharon, 2018). IW24, for instance, worked with nanomaterials for industrial production; to be useful for him was to make new materials that would be beneficial to industry.

I always intended that what I work with should be useful …. By working with a material that we knew industry applied a lot, we framed the research so that it necessarily would be relevant. So, we thought if we combined our focus on this much used material with a focus on nanotechnology, which was new, then somehow there’ll be something useful coming out of it.

IW24 elaborated that a focus on application had been important ever since he started to engage with nanotechnology. Several of the interviewees collaborated with industry partners in their projects to bring in more application-oriented perspectives, to identify social needs, and to facilitate an efficient transition from the research to the application stage. A potential benefit was new jobs.

You don’t change the world, but we have been part of doing research that has led to companies being established, and then people are hired in these enterprises … so in some sense we’ve contributed to creating new jobs. I do not know to what extent one can say that creating new business is important to research, but we have in any case collaborated with industry and developed things they said they needed. (IW20)

Four of the interviewees had been involved in establishing start-up companies based on their research. Following a project-oriented conceptualizing of the common good (rather than an industry-oriented one), they argued that they did this to be able to work more closely with product development than they could within an academic setting. Thus, they explained ‘being useful’ as being engaged with innovations, specifically with innovations that expand networks (Sharon, 2018: 7). For instance, one interviewee explained her motivation for founding her own company by criticizing scientists with a more basic orientation.

[Y]ou’re in cancer research because you want to do something with the disease cancer, and if you then get an opportunity to do something but you don’t bother, or if you’ve an opportunity but don’t pursue it because you think the chance of success is too slim, then you stay at the basic science level. Then you don’t have a goal of contributing [to society], that is my opinion. (IW23)

IW23 stressed that starting a company in a medical field was a long, tedious and expensive process with a high risk of failure. She did not expect the financial gain to be high if they succeeded. A scientist working with nanomaterials articulated a similar sentiment but with a different concern: ‘As scientists, we *do* have the responsibility of trying to work for sustainable development’ (IW5).

Working close to practical applications made it easier for scientists to describe the usefulness of their research. However, some interviewees argued that basic research also was useful, explaining the benefits in other terms. Often, these scientists emphasized that the only thing they could properly plan for was to work hard and provide incremental knowledge gains. Usually, their perspectives on how their research could benefit society were quite abstract. A synthetic biologist expressed it like this:

There’s a problem out there and we know it’s out there, and we’re trying to kind of contribute with our share to maybe solve it. … [W]e can claim to achieve incremental gains of knowledge. Whether this translates into a great drug that saves the world, which would be great for society? It might work, or it might not work. Most probably, it won’t work, right, but this is what science is about, right? (IW31)

Here, IW31 claimed that his research was curiosity-driven but not disassociated from concerns about benefits. Several of those who worked on basic science projects provided similar arguments. They argued that publishing work that others cited and used was the best way of being useful. In this way, they offered a framing of usefulness in terms of providing usable knowledge. On a similar note, IW34 argued that it was a mistake to expect that all scientists should be useful in the same way.

Thus, providing benefits to society was a shared value and an important feature of these scientists’ imagined scientist. The interviewees considered being useful a crucial – possibly the most important – motivation for scientists to do science. They wanted their research to be socially relevant and beneficial. This suggests that, with respect to providing benefits to society, the imagined scientist of the scientists was aligned with the construct of the policy documents. However, this was only true at an abstract level, because there were clear differences in the understanding of what it means to be useful. The RRI-related documents of the RCN present usefulness in a general, abstract manner. They emphasize the urgency of producing social goods, but this is often shaped by invoking an innovation imperative that tends to be entangled in a marketization discourse (Pfotenhauer et al., 2019), rather than on solving actual social challenges. In contrast, the interviewed scientists were more concrete but had a wider range of explanations, including how social benefits could be uncertain achievements that might take a long time to realize.

Accordingly, the imagined scientist of the interviewees was not deficient regarding the commitment to contribute benefits to society. However, this commitment was heterogeneous because it included multiple ways of being useful. The imagined scientist could engage with patients, with grand challenges such as sustainability, with industry and innovations, and with improving knowledge that could be beneficial in a long-term perspective.

### Reflexive competence and knowledge about complex science-society relations

The imagined scientist emerging from the analyzed science policy documents was deficient with respect to practices of acquiring knowledge of, reflecting about and engaging with science-society relationships. The documents’ imagined scientist also had potential for improved observance of ethical and environmental concerns. We asked our interviewees to identify ethical, legal and social aspects of their research and to tell us how they managed such concerns. Apart from references to HSE (health, safety and environment) and animal welfare, a frequent first response of the interviewees was a claim that their research was on safe grounds. However, most of them found it hard to elaborate on concrete concerns regarding risks to society. They explained that they had established their projects to solve a problem that they believed was important to society. Thus, many found it hard to see how their imagined scientist’s work could produce any negative impact if HSE rules were followed.

There are no immediate ethical issues that we [scientists] need to consider, just normal lab safety, to make sure people feel appreciated in the workplace, more on that level really … there is nothing problematic about what I’m doing. (IW3)

Thus, the imagined scientist of the interviewed scientists had no deficits in terms of managing ethical, legal, and social concerns.

We saw how the RRI-related policy documents emphasize that scientists ought to learn more about science-society dynamics in order to navigate this area more effectively and with greater social benefits. The interviewees tended to see such knowledge issues in a different light. They argued that they addressed reflection and learning through both internal and informal processes. IW31 was particularly clear about this.

I never have such good discussions about potential risks and ethical problems of the research that we do, as I’ve had with my fellow scientists. So, these things are discussed, and they’re up in the air all the time as a topic … But we don’t discuss this in a formal forum. It isn’t something that’s enforced or regulated, which doesn’t stop us from thinking about it [social responsibility] so in this way, while we do this [reflect] on a regular basis, in a way, because we think it’s interesting and because we think it’s important. We don’t have procedures to prove that we do it. (IW31)

This argument is interesting because it acknowledges the need to acquire knowledge about social responsibility (as the policy documents suggest), but it presents learning and reflecting as shared tasks among colleagues, as a community affair rather than as something that scientists should address individually or as activities that could benefit from interdisciplinary input. Still, in this interview, social responsibility was framed narrowly, in terms of risks and ethics rather than complex science-society relations. Does that mean that the imagined scientist of the scientists is uninformed about these complex dynamics, as the science policy documents imply with respect to their imagined scientist?

In contrast to the generalized knowledge deficit claimed by the science policy documents, the imagined scientist assembled from the interview accounts displays greater heterogeneity concerning this set of questions. Only the biomedical scientists felt that they had experienced the effects of public perceptions of their work. In particular, those engaged with genetic medicine had learned that their research field could be the object of heated public debate, particularly when it involved prenatal genetic screening. Public scrutiny had pushed these interviewees to pursue the virtue of being reflexive with respect to how they communicated their work to the public. For example, IW18 recounted that she had been very nervous before a newspaper interview about a major finding of her research. She was afraid that the journalist would spin the story the wrong way and create a misconception and was concerned about how she could avoid this (cf. Åm, 2019a: 459). Thus, some scientists were quite knowledgeable about how their research was entangled with society. However, they tended to interpret these complex relations as potentially threatening to themselves and therefore in need of control through orchestration, not as a resource for better science.

The biomedical scientists stood out with respect to how they emphasized the ordering of knowledge implied in social responsibility efforts. The synthetic biologist IW31 above displayed a ‘demarcation rationality’, articulating science as a profession that should have a high level of autonomy from other actors (see Glerup and Horst, 2014: 37) and be able to manage concerns within the community. The interviewees from biomedical fields thought differently. They were working with topics such as genetic medicine and their research involved patients or human biomaterial. These scientists were particularly concerned with ethical aspects of their research, which they saw as concrete and present in their everyday life. They explained to us that the formal, mandatory ethical evaluation of medical projects helped them to learn about ethical matters and how to manage them. Thus, in this context, they considered engaging in such interdisciplinary interaction to be a useful virtue.

I had not given it much thought before, but it’s very important that there exists such a process where an ethical committee approves the projects, which also ensures the quality of the projects. It isn’t always that you as a scientist, when your focus is deep into the scientific questions and you’ve spent a long time getting into the scientific side of the topic … it isn’t always that you then see the ethical aspects of what you’re doing and the ethical implications of your work. (IW29)

In such statements, he and other biomedical scientists described the interaction with ethical committees as a learning arena where they got valuable external input.

Other scientists questioned whether there were any strict demarcations between science and society. They emphasized that a concern for and knowledge about social responsibility with respect to science-society relations was an obvious consequence of being a citizen.

We are often husbands and wives, and often we have children and live in the society … We participate in society on the same terms as anybody else and not like a hermit. So, I also want society to develop in a way that is to the best for its citizens. (IW10)

Overall, the interview accounts show that, unlike the construct of the RCN documents, the scientists’ imagined scientist does not lack knowledge about science-society relations and displays no deficit with respect to aligning research with societal needs. Clearly, this difference reflects different ideas about what kind of knowledge is needed and where it should come from, similar to what we observed in the discourses on social benefits. Should we then conclude that the imagined scientist of the scientists does not have any deficits regarding social responsibility?

### Deficits in translating thought into action

When we asked the scientists about potential harm, they were mainly preoccupied with what they understood as the potential *direct* impact of their work. For example, IW3 was aware of critical discussions regarding nanotechnology and nano medicine. During the interview, we touched upon many such issues, for instance how nano implants could enhance cognitive abilities and the way in which humans perceive their surroundings. However, for IW3, these discussions emerged from long-term visions and not from actual achievements of projects. Thus, he did not see these issues as relevant to his current work or as something that he could influence, because his project was too far from any application.

Moreover, IW3 believed his research would be important to medical progress. Thus, it made little sense to spend much time ‘speculating’ on aspects of social responsibility that he believed were not yet present. In this way, IW3’s account shows an interesting difference regarding how he managed positive and negative impacts in his work. While he considered positive effects on a long-term basis, he confined possible harmful impacts by linking them to immediate concerns. His doubts resemble discussions in RRI scholarship on the art and craft of anticipation (e.g. Nordmann, 2014; Selin, 2014; Swiestra and Rip, 2007: 18). For example, IW3 suggested that anticipatory governance requires grounding in technological achievements and so he did not consider it meaningful to anticipate future impacts of basic science, since this would require knowledge of a whole range of unpredictable scientific and technological breakthroughs. The imagined scientist should not be required to speculate.

In general, many interviewees were eager to present themselves and, by implication, their imagined scientist as reflexive. The challenge, they explained, was to translate knowledge of what it meant to be socially responsible into decisions affecting projects. A recurring point of reference was the infamous Manhattan project and the making of the atom bomb. Synthetic biologists used this example to illustrate how difficult it was for them as scientists to anticipate potentially negative impacts of their work. Moreover, they argued that there was always the possibility of abuse by other, more powerful actors, which we may recognize as a familiar trope in emerging technology discussions (e.g. Swiestra and Rip, 2007: 18). This observed ambivalence about distributed agency, they believed, absolved their imagined scientist from a personal responsibility regarding future (ab)use of their research.

This represents another clear instance of poor alignment of the imagined scientists emerging from the two sets of data. As we observed, the RCN documents construct scientists as powerful and able to produce wide-ranging change in society. Consequently, the imagined scientist should feel obliged to include other actors in their project; the lack of such efforts constitutes a deficit. In contrast, the scientists’ arguments showed that their imagined scientist suffered from a deficit of agency, facing greater uncertainty regarding the outcome of the performed research than just ‘a slight surprise of action’ (Latour, 1999: 266–292).

Still, many interviewees showed concern about potentially negative impacts of their work. To mitigate such concerns, they required that other actors should take part in the enactment of responsibility in the development of technology. IW10 argued, for example: ‘Most societal challenges are much more about political and practical problems that concern equality and the distribution of resources rather than [they are] about research practices.’ This does not mean that interviewees such as IW10 ascribed all agency and responsibility of the imagined scientist to others. IW10 was a pioneer of Norwegian DNA research and saw the 1976 Asilomar conference as a telling example of how attention towards science-society relations and the enactment of social responsibility of research were a normal part of being a scientist. He considered the Asilomar conference to be an expression of scientists caring for social responsibility, because it was the scientists themselves who initiated a moratorium while the potential consequences of recombinant DNA technology were investigated.

Like many other interviewees working with medically related topics, IW10 also reflected upon possible effects on the current health care system. He knew that his research could result in very expensive treatments that would require difficult prioritization, but he was unable to describe how such reflections affected his scientific work. Several other interviewees also claimed to pursue the virtue of being reflexive, while finding it difficult to articulate concretely their reflections, to clarify what knowledge they used, and how their reflexive efforts influenced their work. Thus, their imagined scientist faced challenges when trying to be reflexive about social responsibility but did not lack a willingness to consider such issues. When we asked them to respond to concerns about the need to anticipate and take responsibility for possible negative future impacts of their work, concerns voiced in the policy documents, these interviewees resisted. The implication was that their imagined scientist had no important knowledge deficit regarding what it meant to be a socially responsible scientist but lacked knowledge and control of future impacts of the research.

In this section, we have shown that the imagined scientist of the scientists importantly enacted a research practice aimed at being useful, ethical, rule following and reflexive. Moreover, in their discursive constructs the interviewed scientists – other than the biomedical researchers –resisted the claim of the RCN documents that scientists have a knowledge deficit with respect to social responsibility virtues and how to pursue them. The biomedical interviewees responded somewhat differently, though without remaking the imagined scientist. Bioethics was an integrated part of their scientific practice. Thus, they found it useful to collaborate with ethics committees, but without perceiving this as caused by a knowledge deficit on their part. Overall, the only deficit of the imagined scientist of the scientists concerned a lack of agency to control changes emanating from their research. However, the interviewees did not propose any strategies to amend the latter situation. The problem was not experienced as pressing and as beyond the imagined scientist’s sphere of influence.

## Conclusion: Deficit trouble

The imagined scientist concept makes visible important assumptions implicit in science policy. The discursive construct is a necessary precondition for articulating such policy. At the same time, it has consequences for how scientists perceive science policies. We have used the concept to describe how two sets of actors perceived scientists’ deficits related to the practice of social responsibility and knowledge about science-society relations. We have done this in a symmetric fashion, without assessing the validity of any of the two constructs or claiming that one is better than the other. Table 1 summarizes the main findings, using the dimensions of deficit logics introduced by Pfotenhauer et al. (2019).

**Table 1.** Main features of the deficits of the two imagined scientist constructs.

Features	Version of science policymakers	Version of bio- and nanotechnology scientists
Problem diagnosis	The virtues of acting ethically and avoiding harm are important but providing benefits to society and anticipating future effects of research are more strongly emphasized. At the same time, the latter are not sufficiently pursued by the imagined scientist. Knowledge deficits are diagnosed with respect to assumptions about scientists’ lack of understanding of the complexity of science-society relations.	There is a deficit of agency with respect to how social responsibility can be cared for by whom.
Proposed solution	Deficits should be covered through collaboration with external actors.	The agency deficit was not seen as pressing or within the imagined scientist’s scope of action.
Source of expertise considered legitimate to address deficit	The imagined scientist can improve without outside intervention if disciplining mechanisms (grant proposal schemes) are in place and scientists comply with the RRI goals.	The imagined scientist has a deficit related to ethics in the biomedical area, which is met by collaborating with ethical committees.
Social order	In the final instance, the imagined scientist should enact social responsibility. Culture is not important. Practice is emphasized with respect to performing social responsibility but not the doing of science.	More powerful actors should act responsibly; politics and regulations are important for risk governance. The imagined scientist is embedded in the culture and practice of doing science.
Success criteria	Remains nebulous.	Not relevant because of lack of experienced problems that invite proposals of solutions.

Table 1 shows that the two versions of the imagined scientist are not aligned. The dissonance – what we see as ‘deficit trouble’ – needs further consideration. At first sight, there is an abstract alignment in the sense that the two constructs share a commitment to being socially responsible, including providing benefits to society and being ethical and reflexive. However, when we analyzed the documents and the interviews more closely, we found that they diverged considerably in their understandings of the challenges involved in the enactment of social responsibility. Thus, they articulated incompatible deficits in their respective version of the imagined scientist.

The lack of alignment of the two constructs was most outspoken with respect to features emanating from the discourses related to societal benefits of research and knowledge of science-society relations. The RCN documents insist that society needs to reap greater and more rapid benefits from research, framing this deficit without considering differences between basic and applied research. In contrast, all the interviewed scientists claimed to be engaged in useful research, most emphasizing that being useful was an important personal and professional motivation for being a scientist. Thus, they contradicted the deficit claimed by the RCN documents. Moreover, in particular those working with basic research disagreed with short-term, market-oriented justification of worth, suggesting the need for an extended timeframe for benefits to be recognized.

In the context of STS and RRI scholarship, the reflexivity deficit argued in the RCN documents and the power deficit described by the interviewed scientists deserve further discussion. The claim of a reflexivity deficit is not grounded in the kind of STS literature (e.g. Knorr-Cetina, 1999; Latour, 1987; Traweek, 1988) that has analyzed the cultures and practices of science. Rather, it echoes RRI scholarship (e.g. Owen at al., 2012; Stilgoe et al., 2013), which in turn takes inspiration from STS research on science-society relations that has approached the issue from the perspective of public engagement. In particular, Wynne (e.g. 1993; see also Rommetveit and Wynne, 2017; Welsh and Wynne, 2013) has criticized institutions of science (including science policy) for lack of reflection regarding the power exercised by science/scientists in many situations, the way research may cause new risks, and the complexities of public engagement with science.

However, the understanding of the reflexivity deficit in the RRI framework document of RCN (n.d.) is substantially more limited in its rather instrumental focus on scientists. They are asked to improve their reflection about future outcomes of their research and how results more quickly can be commercialized, all in a risk-reducing manner. It sidesteps the critique of science of Wynne and others about a lack of reflection about the power of science and scientists. This critique also emphasizes the responsibility of institutions rather than individuals, and it is interesting as a counterpoint to the claimed power deficit of the imagined scientist of the interviewees; that it is other actors who shape the future impact of their work. Their imagined scientist is mainly a cog in a large wheel. This view may not be incompatible with Wynne’s argument, when we notice the important difference between a focus on individual scientists and on the institutions of science. The RCN documents acknowledge the role of institutions, including itself, to engage in social responsibility in science-society relations. However, in the final instance, the institutional accountability is delegated to the imagined scientist of the documents. It is the scientists who are asked to take the lead in the enactment of social responsibility of science.

Overall, our findings regarding the imagined scientist of our interviewees suggest that it is misleading to describe scientists in general as unreflexive (McCarthy and Kelty, 2010; Kjølberg and Strand, 2011). Rather, the interviewees emphasized reflexivity. While this activity could be improved, the deficit argument in the RCN documents does not communicate well with the self-understanding of the scientist and may thus be unhelpful. This is a general problem with deficit thinking, which is well known in the area of public engagement with science (e.g. Welsh and Wynne, 2013).

There are also other important implications of the lack of alignment between the discursive constructs made in the two domains. To begin with, the imagined scientists of RRI’s epistemic policy communities (consisting of both scholars and administrators) unfortunately often are too weakly anchored in empirical research about what scientists do and the context they work in. Thus, the constant, active interweaving of science and society is overlooked, even as it shapes and orchestrates the processes of knowledge formation and knowledge sharing. The result is that RRI policies tend to neglect the role of the working conditions of academics and the political economy of science shaped by the innovation imperative (Pfotenhauer et al., 2019) and the increased commercialization of science (Mirowski, 2011). In turn, this may hamper the enactment of responsible research (Åm, 2019b; Felt, 2017; Glerup et al., 2017).

It may be seen as a paradox that the imagined scientist of the scientists is not constrained in the pursuit of social responsibility by working conditions either. However, this reflects the belief of the interviewed scientists that in general, they acted sufficiently responsible – perhaps also echoing Sakslind and Skarpenes’ (2014) observation that Norwegian middle-class morality is characterized by ‘the socially responsible citizen’. Thus, the lack of alignment suggests that there exist robustly different ontologies regarding the performance of science. In turn, this contributes to the deficit trouble, resulting in disagreements between science policymakers and practicing scientists about how science should be reformed. These disagreements may cause a stalemate in further reform efforts and need to be taken into further consideration.

The ‘imagined scientist’ is a sensitizing concept (Clarke et al., 2018) for further studies of science governance across a range of topics. It facilitates the identification of different deficit logics (Pfotenhauer et al., 2019) and how they perform in science policy. For example, it can be helpful in the analysis of gender balancing in universities (Moratti, 2020), of new public management practices at universities (Sørensen, 2019), of excellence frameworks (Flink and Kaldewey, 2018), and of academic valuation practices (Fochler and Sigl, 2018). Not the least, it would be interesting to study the imagined science policymakers of policymakers and of scientists to see if we would observe a parallel kind of deficit trouble.
